# Exercise ventilatory inefficiency may be a specific feature of patients with acromegaly

**DOI:** 10.1007/s11102-025-01585-2

**Published:** 2025-10-16

**Authors:** Gianluigi Dorelli, Massimo Venturelli, Giulia Sartori, Alberto Fantin, Mattia Cominacini, Michele Braggio, Luca Dalle Carbonare, Elia Piccinno, Maria Vittoria Davì, Federico Schena, Ernesto Crisafulli

**Affiliations:** 1 Department of Neuroscience, Biomedicine and Movement Sciences, University of Verona, Verona, Italy; 2 Respiratory Medicine Unit, Department of Medicine, University of Verona and Azienda Ospedaliera Universitaria Integrata of Verona, Largo L.A. Scuro, 10, Verona, 37134 Italy; 3 Department of Pulmonology, S. Maria della Misericordia University Hospital, Udine, Italy; 4 Department of Engineering for Innovation Medicine, University of Verona, Verona, Italy; 5 Department of Emergency Medicine, Integrated University Hospital of Verona, Verona, Italy; 6 Endocrine Unit, Pederzoli Hospital, Peschiera del Garda (Verona), Italy

**Keywords:** Acromegaly, Ventilatory inefficiency, Cardiopulmonary exercise testing, Growth hormone

## Abstract

**Purpose:**

Reduction in exercise capacity and peak oxygen uptake (V̇O_2_) are common in acromegaly, but ventilatory alterations during exercise remain unstudied. We evaluated the exercise ventilatory response in terms of efficiency in patients with acromegaly.

**Methods:**

We prospectively evaluated 10 patients with acromegaly in a phase of disease control. To minimise confounders related to ventilatory response and anthropometric differences, acromegalic patients were matched with 10 control subjects according to age, body mass index, and body surface area. Chronic diseases, any cardio-respiratory condition likely to alter ventilatory response, and evidence of cardiac dysfunction were excluded. Pulmonary function tests and maximal cardiopulmonary exercise testing were performed. Ventilatory response was assessed via minute ventilation (V̇_E_)/carbon dioxide(CO_2_) output slope (V̇_E_/V̇CO_2slope_) and end-tidal carbon dioxide pressure (PETCO_2_). Exercise ventilatory inefficiency (EV*in*) was defined according to reference values for healthy individuals.

**Results:**

No alterations in lung function were observed in either group. Compared with controls, patients with acromegaly showed lower power output and V̇O_2_ at peak (1.38 ± 0.20 vs. 1.90 ± 0.64 L·min^−1^; *p* = 0.033). Breathing patterns were similar to those of controls, but acromegalic patients exhibited higher values of V̇_E_/V̇CO_2slope_ (31.8 ± 3.7 vs. 28.0 ± 2.7; *p* = 0.018), lower PETCO_2_ at peak (31.1 ± 5.3 vs. 36.9 ± 3.0 mmHg; *p* = 0.008), and a greater prevalence of EV*in* (60% vs. 10%; *p* = 0.019). No correlations emerged between ventilatory inefficiency and biochemical or clinical variables.

**Conclusion:**

Patients with acromegaly display ventilatory inefficiency during exercise. These findings suggest subclinical alveolar-capillary impairment or altered CO_2_ chemosensitivity. Exercise testing may help unmask these abnormalities, supporting its role in risk stratification and rehabilitation planning.

## Introduction

A reduction in exercise capacity and peak oxygen uptake (V̇O_2_) is common in patients with acromegaly, even during the biochemical control of the disease [1, 2]. However, no documented alterations related to ventilatory constraints or excess ventilation during exercise have been reported. The steepness with which ventilation (V̇_E_) rises with respect to carbon dioxide (CO_2_) production (V̇CO_2_) characterises a measure of the ventilatory efficiency related to an abnormal ventilatory response to exercise. It is commonly used to clarify the unclear aetiology of the dyspnea symptom and to identify patients with a worse prognosis in several chronic cardiopulmonary diseases, independent of V̇O_2_ [3]. While impairment in cardiac and skeletal muscle accounts for much of this long-term limitation, the excess of growth hormone (GH) and insulin-like growth factor 1 (IGF-1) is well-known to have a systemic impact [1, 2, 4]. Prior reports suggest modest pulmonary alteration in acromegaly, consisting of elevated lung volumes, increased arterial partial pressure of CO_2_ [5], and a potential reduction in diffusing capacity (DL_CO_) [6, 7]. These subclinical alterations led us to hypothesise a potential underlying ventilatory abnormal response during exercise that could impact tolerance in patients with a biochemical control beyond cardio-muscular limitation.

The rarity of the disease and the frequent co-occurrence of upper airway abnormalities make it a challenging task to interpret the literature on exercise ventilation [7, 8]. In addition, to complicate the interpretation, comparing exercise ventilatory responses in these patients may be confounded by bone and soft tissue enlargement associated with the disease. Such anthropometric differences can obscure the contribution of the disease itself unless control groups are matched for anthropometric characteristics such as body mass index (BMI) and body surface area (BSA) [7, 8].

## Methods

At the Respiratory Medicine Unit of the University of Verona and Azienda Ospedaliera Universitaria Integrata of Verona (Italy), we therefore prospectively examined gas-exchange responses to maximal exercise in a highly selected cohort of acromegalic patients in biochemical control [9]. To characterise whether ventilatory alterations were acromegaly-specific, we also selected a control group. These subjects were recruited in the context of the Sports Medicine Unit of the same hospital, when they referred for a non-competitive sports medical certification for participation in amateur sports, before the beginning of sports activities. These subjects were sedentary and without clinical signs of any cardio-respiratory or metabolic significant chronic disease (apparently healthy). To minimise confounders on ventilatory response, adults aged 18–70 years with BMI 18–35 kg·m^−2^ and either clinical and biochemical control (IGF-1 ≤ 1.0 × upper limit of normal, ULN) [8], as well as moderate-to-good quality of life (Acromegaly Quality of Life Questionnaire, AcroQoL ≥ 60) were included [10]. In both acromegaly and control group, therefore, we excluded chronic diseases (except controlled hypertension), any cardio-respiratory condition likely to alter ventilatory response [11], and evidence of cardiac dysfunction (left ventricular ejection fraction < 50%, left ventricular mass index ≥ 115 g·m^−2^ men/≥95 g·m^−2^ women, or diastolic dysfunction >grade I) [12]. The control group was matched according to the predefined parameters of age (± 5 years), BMI (± 10 kg·m^−2^), and BSA (± 0.20 m^2^). Both groups consisted of sedentary subjects who had not engaged in regular physical activity in the past five years.

All participants underwent pulmonary function testing and cardiopulmonary exercise testing (Quark CPET, Cosmed Srl, Rome, Italy) on a cycle ergometer (E100, Cosmed Srl, Rome, Italy) following American Thoracic Society (ATS)/American College of Chest Physicians (ACCP) standards, with a ramp protocol of 10 to 25 W·min^−1^ increments to achieve exhaustion between 8 and 12 min [13, 14]. Ventilatory thresholds, evaluated at the anaerobic threshold (AT) and at the respiratory compensation point (RCP), were determined via ventilatory equivalents and V-slope methods [14]. We measured work rate, V̇O_2_, ventilation (V̇_E_), V̇CO_2_, tidal volume (V_T_), respiratory rate (RR), end-tidal CO_2_ pressure (PETCO_2_), oxygen saturation, and ventilatory efficiency indices (V̇_E_/V̇CO_2slope_, nadir, and AT value). Exercise ventilatory inefficiency (EV*in*) was defined following V̇_E_/V̇CO_2slope_ reference values according to *Sun et al.* [11]. Statistical analysis was performed using Jamovi (Version 2.3.21.0) and included the Shapiro-Wilk test for normality and between-group comparisons using the χ² test, or independent *t-*test or Mann-Whitney U-test as appropriate. The institutional ethics committee approved the study, and all participants gave written informed consent.

## Results

We enrolled 10 patients with acromegaly (seven females) matched with 10 controls (six females). Key general characteristics and cardiopulmonary exercise test (CPET) findings are summarised in Table 1. No deficit in lung function was shown in either group. Among the acromegaly patients, 20% had hypopituitarism and 10% had prolactinoma. A history of pituitary surgery was reported in 60%, radiotherapy in 20%, and ongoing pharmacological treatment in 70% (60% monotherapy, 10% combination therapy). Mean IGF-1 was 0.75 ± 0.19 × ULN, GH 1.35 ± 1.70 µg·L^−1^, and AcroQoL 87.5 ± 11.5 (patients in stable disease with a good quality of life). Two patients (20%) with acromegaly had obstructive sleep apnoea.

**Table 1:** General and cardiopulmonary exercise characteristics of acromegalic patients and control subjects

	Acromegalic patients (*N* = 10)	Control subjects (*N* = 10)	*p*-value
General characteristics			
Females, n (%)	7 (70)	6 (60)	0.639
Age, years	55.8 ± 15.2	53.9 ± 9.8	0.744
Current or former smokers, n (%)	5 (50)	7 (70)	0.174
Arterial hypertension, n (%)	1 (10)	0 (0)	0.305
BMI, kg·m^−2^	25.6 ± 4.4	23.9 ± 2.6	0.307
BSA, m^2^	1.79 ± 0.14	1.75 ± 0.16	0.531
Pulmonary Function			
FVC, liters	4.50 ± 1.1	4.05 ± 1.06	0.343
FEV_1_, liters	3.60 ± 0.89	3.26 ± 1.03	0.434
FEV_1_/FVC, %	80.2 ± 7.8	80.0 ± 5.7	0.948
Exercise Capacity			
Peak Power Output, Watts	101.3 ± 18.7	155.8 ± 67.5	**0.024**
V̇O_2_, mL·min^−1^·kg^−1^			
Rest	5.5 ± 2.2	6.5 ± 3.7	0.470
Peak	19.8 ± 3.5	27.5 ± 6.3	**0.003**
V̇O_2_ at peak, L·min^−1^	1.38 ± 0.20	1.90 ± 0.64	**0.033**
Heart rate, beats per minute			
Rest	78 ± 14.9	61.2 ± 15.9	**0.030**
Peak	147.9 ± 23.5	153.8 ± 15.5	0.516
Ventilatory Response			
V̇_E_, liters			
Rest	14.2 ± 2.7	14.7 ± 8.2	0.853
AT	28.1 ± 4.7	31.3 ± 12	0.446
RCP	46.9 ± 7.3	54.8 ± 18.4	0.221
Peak	66.7 ± 15.1	76.4 ± 28.7	0.357
V̇_E_ ·BSA^−1^, L· m^−2^			
Rest	8.0 ± 1.8	8.5 ± 5.1	0.779
AT	15.8 ± 3	17.6 ± 5.8	0.381
RCP	26.3 ± 4.6	30.8 ± 7.9	0.137
Peak	37.7 ± 10.1	43.1 ± 14	0.338
V_T_, liters			
Rest	1.03 ± 0.40	0.76 ± 0.18	0.063
AT	1.29 ± 0.25	1.52 ± 0.49	0.206
RCP	1.85 ± 0.65	2.05 ± 0.64	0.593
Peak	1.92 ± 0.45	2.14 ± 0.78	0.376
Respiratory rate, breaths per minute			
Rest	16 ± 6.9	19.1 ± 7.7	0.353
AT	22.3 ± 4.4	20.9 ± 5.4	0.509
RCP	27 ± 7.2	26.1 ± 5.5	0.749
Peak	35.9 ± 9.8	37.5 ± 9.3	0.716
PETCO_2_, mmHg			
Rest	27.8 ± 4.8	31.6 ± 3.2	0.051
AT	37.2 ± 4	42.7 ± 3.7	**0.005**
RCP	36.2 ± 3.3	42.4 ± 2.2	**< 0.001**
Peak	31.1 ± 5.3	36.9 ± 3	**0.008**
SpO_2_, %			
Rest	97 ± 1.5	97.5 ± 1.1	0.507
Peak	96.2 ± 4.4	97.2 ± 0.8	0.492
Ventilatory Efficiency			
V̇_E_/V̇CO_2AT_	32.1 ± 3.8	27.4 ± 2.7	**0.004**
V̇_E_/V̇CO_2slope_	31.8 ± 3.7	28.0 ± 2.7	**0.018**
V̇_E_/V̇CO_2nadir_	30.2 ± 3.6	25.0 ± 2	**< 0.001**
EV*in*, n (%)	6 (60)	1(10)	**0.019**

The data are reported as the number of patients (percentage) or mean ± standard deviation. In bold are reported significant values

BMI indicates body mass index, BSA, body surface area; FVC, forced vital capacity; FEV_1_, forced expiratory volume in the first second; V̇O_2_, oxygen uptake; V̇_E_, minute ventilation; AT, anaerobic threshold; RCP, respiratory compensation point; V_T_, tidal volume; PETCO_2_, end-tidal partial pressure of carbon dioxide; SpO_2_, pulse oximetry oxygen saturation; V̇_E_/V̇CO_2_, V̇_E_ to carbon dioxide output-V̇CO_2_ ratio; _AT_, anaerobic threshold; _nadir_, lowest value of the V̇_E_/V̇CO_2_; EV*in*, exercise ventilatory inefficiency

Compared with controls, patients with acromegaly had a lower peak power output and V̇O_2_ at peak. Also, the absolute value of V̇O_2_ at peak was different (1.38 ± 0.20 vs. 1.90 ± 0.64 L· m^−1^; *p* = 0.033). Despite the similar pattern in exercise ventilation with no substantial difference in V_T_ and RR response, acromegaly patients had higher values of V̇_E_/V̇CO_2slope_, and lower values of PETCO_2_. Patients with acromegaly have prevalently EV*in* in comparison to the control group (60% vs. 10%). No correlations were observed between exercise ventilatory variables (or inefficiency) and general, functional, biochemical or any data reported in patients’ clinical history with acromegaly.

## Discussion

Our data show, for the first time, that patients with acromegaly, even those in the phase of disease control and without cardiac or pulmonary impairment, may exhibit alterations in ventilatory efficiency during exercise, regardless of evident changes in breathing pattern and body size. In these patients, although modifications in exercise ventilatory patterns may be rationally based on upper airway changes, early fatigue of the respiratory muscles due to myopathy, and geometric changes in the rib cage [4, 7], these alterations should hypothetically be present in patients with an advanced disease, when cardiopulmonary involvement is often present. The exercise-induced ventilatory alterations, suggesting an increase of the in dead space ventilation and a ventilation-perfusion mismatch [15], may be reported in our patients in a phase of disease control and without cardiopulmonary impairments, as a sign of an enduring and specific subclinical impairment of the pulmonary circulation and alveolar-capillary membrane, similar to other pulmonary conditions, for example, in post-COVID subjects [15, 16]. Indeed, although with a normal lung function measured by spirometry, post-COVID subjects may show elevated V̇_E_/V̇CO_2slope_ and low PETCO_2_ [16], interpreted as evidence of pulmonary hypertension, persistent endothelial injury, microthrombotic phenomena, and microvascular damage in the alveolar-capillary membrane since they were related to the positive correlation between alveolar-arterial gradient during exercise and the levels of D-dimer plasma concentration during hospitalisation [17]. Acromegalic lungs show similar evidence of altered alveolar-capillary structure, such as increased lung volumes, possible alveolar hyperplasia or hypertrophy, but also reduced DL_CO_, raising the possibility of microvascular rarefaction or increased perfusion distances [5–7]. The heterogeneity of pulmonary perfusion during exercise may therefore explain the presence of EV*in* in patients with acromegaly, with subclinical manifestations similar to post-COVID subjects. Moreover, since chronic IGF-1 excess may induce vascular endothelial dysfunction, systemic microvascular injury, and oxidative stress, it may be plausible that acromegalic patients may have an alteration of the pulmonary circulation homeostasis [18].

A second, not mutually exclusive, explanation is that EV*in* may indicate an abnormal CO_2_ chemosensitivity resulting from chronic exposure to GH/IGF-1 excess [19], which could explain the evidence of higher arterial CO_2_ and bicarbonate levels in patients with acromegaly compared to controls [5, 8]. In this context, it has been demonstrated that in acromegalic patients, the extent of GH/IGF-1 excess correlates positively with an exaggerated ventilatory response to CO_2,_ which may be the physiological basis for central apnoea’s development [20]. However, the effects of chronic GH/IGF-1 excess on the central respiratory centre remain poorly defined and warrant targeted investigation.

Our study’s strengths include the strict inclusion/exclusion criteria, which help reduce confounding in the ventilatory response. Moreover, the possibility of having control subjects matched to the characteristics of patients with acromegaly may well contextualise our specific CPET findings. In any case, the principal limitation is the small sample size, inherent to the rarity of acromegaly [8]; this aspect may be the reason why no correlations were observed between ventilatory variables and disease-related variables. Accordingly, these results should be viewed as preliminary observational data and not generalised to all patients with acromegaly. Moreover, another significant limitation is the lack of a bone morphometric evaluation, especially for vertebral fractures [21] that may compromise chest wall mechanics and potentially contribute to the exercise ventilatory alterations observed during exercise. Finally, we lack a measure of the DL_CO_ to confirm the alteration in the resting alveolar-capillary membrane of these patients. Nevertheless, the consistency of greater EV*in* prevalence and lower PETCO_2_ supports the hypothesis of an exercise-unmasked respiratory involvement in acromegaly, even when the disease is controlled and cardiac function is preserved [15]. Recognising this subclinical respiratory involvement through exercise testing, which evaluates maximal capacity and ventilatory efficiency, may refine risk stratification and inform rehabilitation strategies, including targeted aerobic conditioning and, where appropriate, evaluation for pulmonary vascular disease or diffusion impairment. Larger, multicentre studies that increase the number of patients considered should validate these observations, delineate mechanisms, and assess responsiveness to therapy and rehabilitation programs. Moreover, it would be interesting to compare controlled and uncontrolled acromegalic patients to assess the relationships between the exercise-ventilatory alterations and the disease severity.

## Data Availability

The datasets used and/or analysed during the current study are available from the corresponding author on reasonable request.

## Declarations

### Clinical trial registration

This study was not registered.
